# Mild Diabetes Models and Their Maternal-Fetal Repercussions

**DOI:** 10.1155/2013/473575

**Published:** 2013-06-26

**Authors:** D. C. Damasceno, Y. K. Sinzato, A. Bueno, A. O. Netto, B. Dallaqua, F. Q. Gallego, I. L. Iessi, S. B. Corvino, R. G. Serrano, G. Marini, F. Piculo, I. M. P. Calderon, M. V. C. Rudge

**Affiliations:** Laboratory of Experimental Research on Gynecology and Obstetrics, Department of Gynecology and Obstetrics, Botucatu Medical School, Universidade Estadual Paulista (Unesp), 18618-970 Botucatu, SP, Brazil

## Abstract

The presence of diabetes in pregnancy leads to hormonal and metabolic changes making inappropriate intrauterine environment, favoring the onset of maternal and fetal complications. Human studies that explore mechanisms responsible for changes caused by diabetes are limited not only for ethical reasons but also by the many uncontrollable variables. Thus, there is a need to develop appropriate experimental models. The diabetes induced in laboratory animals can be performed by different methods depending on dose, route of administration, and the strain and age of animal used. Many of these studies are carried out in neonatal period or during pregnancy, but the results presented are controversial. So this paper, addresses the review about the different models of mild diabetes induction using streptozotocin in pregnant rats and their repercussions on the maternal and fetal organisms to propose an adequate model for each approached issue.

## 1. Introduction

Diabetes mellitus (DM) is considered a chronic disease characterized by hyperglycemia resulting from insulin resistance and/or insulin secondary deficiency caused by failure in beta cells (*β*) pancreatic [1]. DM1 is an autoimmune disease in which there are deficiency of insulin and loss of control of blood glucose, caused by the destruction of pancreatic *β* cells mediated by T cells [2]. DM2 is characterized by *β*-cell dysfunction and decreased insulin action in some tissues [3]. Another classification is the gestational DM, which occurs by glucose intolerance with variable magnitude. It is first diagnosed during pregnancy and may or may not persist after delivery [4]. 

Human studies that explore mechanisms responsible for changes caused by diabetes are limited not only for ethical reasons but also by the many uncontrollable variables (diet, socioeconomic factors, nutrition, and genetic factors) that can alter the intrauterine environment and increase congenital malformations. So there is a need to develop a suitable experimental model [5]. The use of animal models provides an essential tool for investigating the molecular mechanisms that control cell growth. The maternal-fetal interface is no exception. Although there are some differences in the organization of rodent versus primate maternal-fetal interface, there are many similarities in the functions and in cell lines that compose it. The induction of experimental diabetes by cytotoxic drugs such as beta-streptozotocin (STZ) is well characterized [6]. Depending on the animal strain used, dose, route of drug administration, and the life period in which STZ is administered in rats, different glycemic intensities are achieved: severe diabetes (blood glucose superior to 200/300 mg/dL) [7–13] or mild diabetes (glycemia between 120 and 200/300 mg/dL) [14–18].

An ideal experimental model of gestational diabetes should have normal glycemia levels before gestation but glucose intolerance and impaired insulin secretion and/or function after midpregnancy, which leads to alterations in both glucose and lipid metabolism in the mother and consequently in the fetus [19]. Therefore, mild diabetes is employed in experimental studies to reproduce blood glucose levels as observed in human pregnancy. 

This paper addresses a review about the different models of mild diabetes induction in rat pregnancy. This review includes repercussions on the endocrine pancreas, early embryo development, reproductive performance, oxidative stress, structural analysis of the extracellular matrix and urethral striated muscle, and transgenerational effects.

## 2. Models of Mild Diabetes Induction and Repercussions on the Maternal and Fetal Organisms

### 2.1. Endocrine Pancreas

Studies about the effects of diabetes in the endocrine pancreas address *β*-cell regeneration by different mechanisms like neogenesis, proliferation, and transdifferentiation of these cells in animals. Neogenesis consists in generation of new cells from undifferentiated cells. When a differentiated cell undergoes transgression and becomes another type of cell, this is termed transdifferentiation. Proliferation consists in cell replication itself [20, 21]. 

The regeneration of *β* cells in STZ-treated pancreata has already been demonstrated in neonatal rats [22]. To evaluate the relationship among the morphology of the pancreatic islets, plasmatic glucose, and insulin, Bonner-Weir et al. [23] intraperitoneally induced diabetes by streptozotocin administration at a dose of 90 mg/kg on day 2 of life in rats. At day 4 of life, the rats presented hyperglycemia and reduced *β* cells number. At day 10 of life, there were partial recovery of the *β* cells and normoglycemia. However, after six-weeks, return of hyperglycemia and decreased *β*-cell volume were observed, therefore, suggesting that these cells are able to partially regenerate.

 STZ administration (100 mg/Kg, saphenous vein) on the day of birth of the neonate rats [14, 15] caused high mortality (30 to 50%) [14]. These animals showed decreased pancreatic insulin and hyperglycemia (200–300 mg/dL) [14, 15]. Diabetes presented a quick and spontaneous remission observed by gradual increase of pancreatic insulin of these animals [14], however, during adult life, the animals showed a decrease of insulin secretion [15]. It was concluded that the STZ administered in neonatal life causes regeneration, however, there is a partial loss of *β*-cell mass and its function. Thus, it suggests that the STZ administered in newborns can not only be used for experimental diabetes but can also be used as a model for studies on neogenesis and/or proliferation of beta cells [14]. Similarly, Movassat et al. [24] intraperitoneally used the same dose of STZ (100 mg/Kg) and observed three days after treatment that the newborn rats exhibited diabetes, decreased body weight, and reduced *β* cells number. Seven days after administration of STZ, all animals showed increased proliferation of *β* cells, but the group treated with insulin presented a higher number of cell replication. The insulin-favored regeneration of the *β* cells reflects both an increased replication from differentiated *β* cells and an increased neogenesis from precursor/stem cells, with this last pathway being preferentially activated. 

Thyssen et al. [25], Nicholson et al. [26], and Liang et al. [27] used 70 mg/kg STZ intraperitoneally on day 4 of postnatal life of rats, and the hyperglycemia (200–300 mg/dL) was verified two days after induction of diabetes. The animals that received the drug presented increased glycemia and partial destruction of *β* cells [25–27] accompanied by a relative loss of the nucleus islet microvasculature of rats [26]. There was a decrease in pancreatic beta cell mass, but these cells showed rapid regeneration because hyperglycemia was transient. This fact suggests that generation of new islets and restructuring of existing islets may occur [25–27]. Thus, a rapid regeneration that appears to involve the generation of new islets [25], the restructuring of preexisting islets [26], or transdifferentiation of the alpha cell to beta cell was documented [27], but studies indicate that age may limit the mechanism of cell regeneration [25].

Thus, the different studies showed that, regardless of the doses and route of streptozotocin administered in neonatal period, the transient hyperglycemia is resulting from partial regeneration of the beta cell. However, the pathophysiological mechanism involved in this regeneration is unclear.

### 2.2. Reproductive Performance

The hyperglycemia of gestational diabetes can lead to changes in maternal reproductive performance and embry-ofetal development [28]. Perhaps one of the most devastating diabetes complications is diabetic embryopathy, in which the offspring of a mother with diabetes predating a pregnancy presents congenital malformations. These malformations can affect multiple organ systems, including the brain and spinal cord, the heart and major vessel, kidneys, gut, and skeletal structures [29, 30], and result in pre- or postnatal mortality or disability. 

The cellular damage that occurs in differentiated tissues leading to disease may also occur in differentiating embryo tissues but, in addition, malformation may result because signaling caused by excess glucose metabolism interferes with tissue morphogenesis. Because the observations of structures were formed, the factors involved in the malformation appearance cannot be determined. It is important to identify and intermediate in these factors in preimplantation period to prevent fetal malformations.

A unique characteristic of preimplantation development of mammals is that the embryo at this stage is self-regulative; that is, during the first three cleavages, the embryo is highly adaptable and can resist insults with the removal, addition, or rearrangement of its blastomeres [31–33]. Even so, changes that occur in the first stage of embryonic development have an impact on pre-and postimplantation [34]. In addition, initial embryos are able to grow in synthetic culture medium for several days without presenting changes even after being returned to the uterus [35]. Thus, most studies related to diabetes and embryo development use this ability of the embryo as a tool to monitor embryo development in vitro, trying to justify the low implantation rates and the malformation occurrence in diabetic women. As a result, several models are used to explore the pathophysiological mechanisms involved. Although this review has been focused on the models of mild diabetes induction, we studied in vivo model of the mild diabetes, and there are few published articles on the effects/consequences of this type of diabetes at the beginning of embryonic development. Therefore, this section shows an overview of the studies and in vitro models of diabetes with high glycemic intensity (severe diabetes).

Mihajlik et al. [36] used embryos recovered (day 3) from females mice that received different doses of streptozotocin (130 or 160 mg/kg ip), 14–17 days before mating. These embryos were transferred to the environment in vitro and analyzed after 48 hours of cultivation. The embryos from mice that received STZ 130 mg/kg showed delay in cell proliferation, and those from mice given 160 mg/kg STZ exhibited a high degree of degeneration. However, on day 3 of pregnancy, these animals showed similar blood glucose levels to the control group. After glucose overload, both groups missed reestablishing initial glucose levels, suggesting that changes in maternal insulin levels may affect the embryo in the preimplantation resulting in alteration of cellular distribution during cultivation in vitro and in highest dose of beta-cytotoxic drug, inhibiting the progression of embryonic development after the third cleavage which reinforces the idea that the plasticity of embryonic cells decreases as the embryo develops.

The culture of mouse embryos in serum of women with diabetes [37] results in decreased viability, however, caused no developmental delay. Changes in embryonic growth are found in vitro models as in the study proposed by Wyman et al. [34] that involves embryos exposed to high glucose concentrations in vitro at the fertilization period and, after that, transferred to the uterus of control rats or on the model proposed by Pampfer et al. [38], using uterine cells from diabetic rats as component of the culture medium in which the embryos are developed. Fraser et al. [39] exposed embryos to different glucose concentrations and showed that at high concentrations (15.56 and 25.56 mM glucose) the numbers of blastocysts and cells per blastocyst were reduced at the 5th day of pregnancy; however, the apoptosis rate of these cells was unaltered. In contrast, other investigators describe a relation between decreased cellular number and increased apoptosis in embryos exposed to hyperglycemic environment [40, 41]. Furthermore, increased glucose concentration alters the distribution of embryonic cells causing a decrease in the inner cells mass number while causing an increase in trophoectoderm cells number [39].

These in vitro results could explain the low implantation rate and increased losses and embryonic malformations in pregnancy complicated by diabetes. However, when the zygote is transferred from diabetic uterus to healthy uterus, developmental delay occurs in addition to malformations (neural tube, ribs, and abdominal wall defects), although the exposure time in hyperglycemic environment is short. When the zygote is submitted to high glucose concentrations in vitro for the same period and transferred to the uterus of a female healthy, embryos exhibit reduced capacity for development, demonstrated by an increase in the rate of reabsorption and decreased sites of embryo implantation [34]. While in vitro analysis of embryos allows monitoring normal morphology [42], recent studies indicate that culture of embryos can break embryonic epigenetic control mechanisms leading to altered expression of some genes [43, 44], resulting in embryos that respond differently from those which develop in the in vivo environment.

Furthermore, while high concentrations of glucose are used in vitro studies, many researchers have developed models of diabetes in animals to reproduce DM2 and/or DM gestational to better understand the pathophysiology. A single in vivo study has associated the increased number of morulae and increased number of blastocysts at 5th day of pregnancy at lower doses of streptozotocin to rats with moderate diabetes (glycemia between 120 and 300 mg/dL). Changes in early development in vivo demonstrate that embryos arising from rats with moderate diabetes have developmental delay, similar to embryos from rats with severe diabetes. Moreover, when maternal hyperglycemia increases, the embryos show higher apoptosis rate [45]. Since metabolic insults, such as diabetes, can permanently affect the initial development [34], the use of in vivo models can be a way to clarify the possible mechanisms involved in the increased embryonic loss and malformation in type 2 DM.

 Our research group aimed to induce the mild diabetes in laboratory animals. In this regard, female offspring received STZ (100 mg/kg, sc) at first day of life. At adult stage, the Wistar rats presented at term pregnancy increased embryonic losses before and after the implantation process, reduced corpora lutea number and decreased number of live fetuses [46, 47], increased weight and placental index, reduced rates of fetuses with appropriate weights for gestational age, and decreased degree of ossification, showing delay in fetal somatic maturation [46]. Dallaqua et al. [48], using the same model of diabetes induction but with another stain (Sprague Dawley), found a reduction in the number of live fetuses, fetal viability index, and increased number of embryonic deaths. In other study, mild diabetes was induced using STZ (70 mg/kg ip) in female rats on day 5 of postnatal life, and the results showed altered maternal lipid profile, increased oxidative stress, and increased frequency fetal visceral anomalies [49]. The same research group verified similar alteration when STZ was administered at birth (100 mg/kg, sc) and at day 7 of pregnancy (20 mg/kg, ip), confirming that glycemic alterations may impair ovulation and the early stages of embryonic development. Besides, intrauterine growth restriction (IUGR) in the fetuses from these rats was also observed. Regarding the analysis of skeletal fetuses of diabetic rats, the sterna showed abnormal morphology and absent cervical nuclei [50]. 

Another model using STZ (70 mg/kg, ip) on the 5th day of life in female rats showed a reduction in the number of corpora lutea, lower maternal weight due to higher rates of fetuses with small weight for gestational age, and a lower rate of fetuses large for gestational age [51]. In addition, there was an increase in the number of fetuses with visceral abnormalities (hydronephrosis and hydroureter) [18].

 Kervran et al. [52], in order to analyze the development and maturation of *β* cells in fetuses from mothers with mild diabetes, administered different doses of STZ (from 30 to 50 mg/kg, iv) in adult rats before mating and verified that rats showed an increase in placental weight, but not in fetal weight, even with high levels of plasma insulin. These conflicting results could be explained by the shorter duration of pregnancy of the animals and the existence of a reduced fat mass of fetuses compared to human, which does not allow the verification of macrosomia in fetuses of rats with mild diabetes. 

When STZ (37 mg/kg, ip) was administered at day 5 of pregnancy, it was found that diabetic rats presented reduced insulin levels during the last week of pregnancy. Moreover, most of the fetuses presented macrosomia and high levels of plasma glucose and insulin at first day of life [53].

López-Soldado and Herrera [54] used different doses of streptozotocin (25, 30, and 35 mg/kg, iv), at the onset of pregnancy in rats. It was observed that, on the day of birth, the rat newborns of diabetic dams presented elevated plasma insulin and macrosomia. Caluwaerts et al. [55] also tested different single doses of STZ (30, 35, 40, or 50 mg/kg, ip) on the first day of pregnancy and on two other groups of rats with two lower doses of STZ (one given two days prior to mating and one on the first day of pregnancy: from 30 to 20 or from 30 to 30 mg/kg, ip). There was a reduced maternal weight gain during pregnancy in groups 35 and 50 STZ, and only STZ groups 30/30 and 50 showed fetuses with hyperglycemia on the first day of life. On the first day of birth, fetuses of STZ groups 30/30, 40, and 50 were small and showed larger placentas. Only, STZ groups 30, 30/20, in 35 and there was an increase in plasma insulin. 

Some studies cited regarding the induction of diabetes between day 0 and 5 of pregnancy were successful as an ideal model, since the doses and routes of administration resulted similarity in results when compared to gestational diabetes in human, besides the induction period, which is corresponding to the preimplantation, thus avoiding the transfer of *β*-cytotoxic drug (STZ) from mother to fetus through the placenta and its possible influence on the development of the offspring. The experiments that had failed during the same period can be explained by dose and route of administration. In the first case one should take into consideration that high doses in ways which favor the release of the drug over a prolonged period cause lasting action, and in the second case, the administration of a drug intravenously favors this movement directly into the bloodstream, which leads to faster and more potent compared to other routes of administration.

The literature investigations that employ or have employed different models of induction of mild diabetes (blood glucose between 120 and 300 mg/dL) analyzed maternal and perinatal repercussions by vaginal delivery few days after birth, which contributes to the findings of macrosomia in offspring from diabetic dams, due to their greater adipose tissue during this period [54]. However, because of our research group to assess the diabetes effects in dams and fetus by laparotomy, it was evidenced higher percentage of pups with growth restriction, which does not corroborate with clinical diabetic mothers found.

 In general, studies show that the diabetes induction by STZ before and after mating led to an exacerbated hyperglycemia, which features a severe diabetes and not mild diabetes. If we consider that this hyperglycemia in early pregnancy can influence embryonic development, this model should not be used to study maternal reproductive performance. The diabetes induction of STZ at the birth of rats caused glucose intolerance in adulthood and during pregnancy, becoming a model for the study of the effects of mild diabetes in the maternal and embryonic organisms. When the STZ administration was performed on the 5th day of life, our results showed favorable glucose levels for characterization of mild diabetes model. The induction on the 5th day of pregnancy, as established by the research group Merzouk et al. [16], shows excellent results with regard to blood glucose levels and rates of macrosomic newborns. However, these authors did not evaluate the developing embryo, which undertakes the study of reproductive performance.

### 2.3. Oxidative Stress

 Oxidative stress occurs when the production of free radicals is higher than the capacity of the antioxidant defense system. This state is characterized by molecular and structural damage in the cell, such as membranes, DNA, lipid, and protein. Studies have shown that hyperglycemia and pregnancy induce increased production of reactive oxygen [56] and nitrogen species [57]. The first line of defense against oxidative damage is the endogenous enzymatic antioxidants: superoxide dismutase (SOD), catalase, and glutathione system, among them are glutathione reductase (GSH-Rd) and glutathione peroxidase (GSH-Px). There are also nonenzymatic antioxidants that are liposoluble (tocopherols, carotenoids, quinones, and bilirubin) or hydrosoluble (ascorbic acid, uric acid, and proteins bound to metals) [58, 59].

To evaluate the oxidative stress status in mild diabetic models, diabetes was induced by streptozotocin (100 mg/kg sc) in Sprague Dawley rats on the day of birth. At term pregnancy, higher MDA concentration and no change on the SOD and GSH-Px enzymatic activities and concentration of thiol groups was observed, showing an exacerbated oxidative stress [48]. In other study, mild diabetes was induced using STZ (70 mg/kg ip) in female Wistar rats on day 5 of postnatal life. At the end of pregnancy, the mild diabetic rats showed that SOD enzymatic activity was reduced and MDA concentration (lipid peroxidation) was slightly increased, confirming oxidative stress status [18]. Yessoufou et al. [60] showed that the diabetes induced on t day 5 of pregnancy using STZ (40 mg/kg ip) caused mild diabetes on days 12 and 21 of pregnancy, decreased SOD, GSH-Rd, and GSH-Px enzymatic activities, and increased TBARS concentration, showing elevated oxidative stress in diabetic animals. 

Although there are different models to induce mild diabetes, an exacerbated oxidative stress status was found in several studies. However, the existing differences are altered/unaltered scavenger activities in these investigations. 

### 2.4. Morphological Analysis of the Extracellular Matrix and Urethral Striated Muscle

Diabetes mellitus during pregnancy is associated with high urinary incontinence levels and pelvic floor muscle dysfunction, but the exact pathophysiology of urinary incontinence (UI) has not been well characterized. UI is known to be multifactorial in origin, including malfunction of the urethral sphincter [61, 62]. The development of animal models is essential for understanding these events and developing preventive interventions [63].

Based on the hypothesis that the muscle and connective tissue integration are intimately related to urinary continence, diabetic animals have great chances to present changes in these tissues. Therefore, a study with mild diabetes model in pregnant rats that received neonatally streptozotocin (100 mg/kg, sc) was conducted. At term pregnancy, the morphological analysis of the extracellular matrix and urethral striated muscle from urethrovaginal tissues showed that the urethral layers were thin, disorganized, and atrophic and there were more fibrosis replacement and collagen fiber deposition associated to muscle atrophy. In addition to structural analyses, one of the most significant findings was the colocalization of fast and slow fibers and a steady decrease in the proportion of fast to slow fibers by immunohistochemical examination in the mild diabetic pregnant rats [64]. Diabetes is related to accumulation of reactive oxygen species, and tissue ischemia which can interactively or independently contribute to the myopathy causes of skeletal muscle dysfunctions [65, 66]. It is well established that the muscle fibers are able of altering their physiological and biochemical properties according to stimulus which they are submitted, reflecting on amount or type of muscle proteins [67]. These changes are often associated with altered glucose metabolism, diabetes and obesity. Skeletal muscle can adapt to functional and metabolic demands by remodeling with fiber type switches to maintain a normal energy balance and utilization of nutrients [68].

Vascular complications are also associated with the progressive severity of diabetes [69] which showed a significant increase in blood vessels in the urethra of mild diabetic pregnant rats. The literature describes the diabetic time-dependent alterations in macrovascular structure and cellular function, suggesting that one of the underlying mechanisms responsible for diabetes-induced vascular disease is uncoupled mitochondria, with increased electron leak and the generation of reactive oxygen species [69].

The mild diabetic pregnant rats also showed an increased interstitial collagen in the ultrastructural analysis. Glycogen granules, lipid droplets, and numerous mitochondria were apparent in the striated muscle cells [64]. Lipid droplets are not membrane bound, and their number and size may vary considerably among different muscle types. They are frequently associated with mitochondria and sometimes completely encircled by them. Experimental enzyme deficiencies in mitochondrial energy metabolism induced the accumulation of giant mitochondria and numerous lipid droplets. This has led to the suggestion that mitochondria may use lipids as a source of energy for muscular contraction. The increased number of lipid droplets in the diabetic pregnant rats indicates that lipids may be an important energy source for striated muscle [70].

These results indicate that mild diabetes in pregnant rats can lead to time-dependent disorder and tissue remodeling in which the urethral striated muscle and extracellular matrix has a fundamental function [64]. Since this is a unique study, there is no description of a best model or comparison with other models in order to reach a conclusion. These findings show that there is a relation between the changes found in the urethra-vagina tissue that would be similar to which occur in the urethra of diabetic women with UI.

### 2.5. Another Model to Obtain Mild Diabetes

In mammals, the growth and development in fetal intrauterine environment are completely dependent on the nutrients provided by the mother. The maternal requirement of carbohydrates, proteins, and lipids increases considerably during this period [71, 72]. In rats, as in humans, glucose is the main substrate for the development of the fetus, which comes from the maternal circulation. Whereas the insulin is the main growth hormone to the fetus, which is responsible for 50% of fetal weight [73]. 

An inappropriate maternal-fetal food supply or nutrient flow leads to marked changes in maternal metabolism by modifying isolated fetal nutrition. Thus, further adjustments are needed during development in utero environment [74, 75]. Without these adjustments the postnatal growth is impaired. Health conditions in the course of adulthood result from the combination between genotype and phenotype, which begins in the prenatal period. This effect is known as “fetal origins of adult disease” [29, 30, 76]. 

The literature studies show transgenerational effects in animal models of hyperglycemia. When female Wistar rats received STZ (30 mg/kg) on the day of mating, it was observed that these rats presented a mean blood glucose 250 mg/dL at the end of pregnancy. The authors found that maternal hyperglycemia impaired glucose metabolism in subsequent generations [77–81]. The first-generation fetuses showed hyperglycemia, and at adulthood (100 days old) these rats showed normal glucose basal levels and endocrine pancreas morphology. However, when glucose load (glucose infusion) was administered, some animals showed glucose intolerance and others presented mild diabetes during pregnancy [82, 83]. The subsequent generation was affected by unfavorable maternal intrauterine environment, and their fetuses showed hyperplasia of pancreatic islets, beta-cell hyperactivity, hyperinsulinemia, and macrosomia [77]. It was verified that the fetal hyperinsulinemia was due to the low levels of amino acids, which can be explained by higher amino acid uptake by placental unit, which can be coresponsible for the decreased insulin response [83]. According to some authors, the transgenerational effect of diabetes crosses through the generations only by the female offspring. However, even if the male present impaired glucose tolerance, there is no transmission to their descendants.

 When STZ (40 mg/Kg ip) was administered at day 5 of pregnancy, the animals presented glycemic levels from 100 to 300 mg/dL, confirming mild diabetes. At birth, pups from mild diabetic dams presented higher mean weight compared to control pups, confirming macrosomia. The macrosomic fetus presented higher serum insulin, glucose, and lipid levels and maintained accelerate postnatal growth combined with high adipose tissue weight up to 12 weeks of age without being hyperphagic. This metabolic disturbances varied according to age and sex. At 2 months of age, hepatic and serum triacylglycerol levels were higher in macrosomic females than in controls. By 3 months, macrosomic rats (males and females) had development insulin resistance with hyperinsulinemia, hyperglycaemia, and higher serum and hepatic lipids [16]. The same research group demonstrated that fetal obesity is associated with alterations in VLDL lipid fatty acid composition and represents an important risk factor for adult obesity and diabetes [84]. Besides these results, it was observed that obese offspring (3 months) presented higher serum lipoprotein concentration, typical of obese and diabetic woman begins [85].

 To induce mild diabetes, Sprague Dawley rats received STZ (35 mg/Kg ip) at day 5 of pregnancy. The pups born to the mild diabetic dams had higher birth weight (macrosomia), pancreatic insulin content, plasma insulin, and C-peptide concentrations compared to those pups born to control dams. At 6 weeks of age, an accelerated growth in the female rats was associated with higher fat weight. At adulthood, higher plasma insulin and glycemia in macrosomic rats, only the female macrosomic rats, showed abnormal glucose response due to peripheral insulin resistance. In the male rats, a higher plasma insulin concentration in the macrosomic group was associated with a normal plasma glucose response to oral glucose challenge. This study showed that maternal mild diabetes in rats led to fetal hyperinsulinemia and accelerated fetal growth [86]. To study the cross-generation effect of newborn rats in the mild diabetes model, similar methodology was used. The first generation of rats presented glycemic levels from 100 to 300 mg/dL, confirming mild diabetes. These dams had macrosomic pups presenting hyperinsulinemia and accelerated growth. The second-generation macrosomic males and females and nonmacrosomic males and females were mated to obtain the third generation. It was observed that the pups from macrosomic parents presented higher birth weight and plasma insulin levels. At adulthood, these animals presented an accelerated growth and higher fat tissue weight compared the control pups, showing that an abnormal intrauterine metabolic environment can lead to obesity and glucose intolerance across generations in rats [87].

 Another model to induce mild diabetes was employed by our group, who administered STZ (40 mg/Kg iv) in the first generation of adult Wistar rats before the mating period. During pregnancy, these rats presented glycemia higher than 300 mg/dL, characterizing an abnormal intrauterine environment. The offspring from these dams presented glucose intolerance at adulthood, showing impaired repercussions of maternal hyperglycemia [88, 89]. 

 The different models of mild diabetes induction to study the cross-generation effect are important to demonstrate that transgenerational effect from an abnormal intrauterine environment causes metabolic alterations across further generations.

## 3. Conclusion

Many models mentioned to induce diabetes lead to similar characteristics compared to those resulting from human gestational diabetes. Thus, according to the targeted objectives, each researcher should choose the best model of diabetes induction for the study.
